# Survivin gene levels in the peripheral blood of patients with gastric cancer independently predict survival

**DOI:** 10.1186/1479-5876-7-111

**Published:** 2009-12-22

**Authors:** Loris Bertazza, Simone Mocellin, Alberto Marchet, Pierluigi Pilati, Joseph Gabrieli, Romano Scalerta, Donato Nitti

**Affiliations:** 1 Department of Oncological & Surgical Sciences, Section of Clinica Chirurgica 2, University of Padova, via Giustiniani 2, 35128, Padua, Italy; 2 Istituto Oncologico Veneto IRCCS, via Gattamelata 64, 35128, Padua, Italy

## Abstract

**Background:**

The detection of circulating tumor cells (CTC) is considered a promising tool for improving risk stratification in patients with solid tumors. We investigated on whether the expression of CTC related genes adds any prognostic power to the TNM staging system in patients with gastric carcinoma.

**Methods:**

Seventy patients with TNM stage I to IV gastric carcinoma were retrospectively enrolled. Peripheral blood samples were tested by means of quantitative real time PCR (qrtPCR) for the expression of four CTC related genes: carcinoembryonic antigen (CEA), cytokeratin-19 (CK19), vascular endothelial growth factor (VEGF) and Survivin (BIRC5).

**Results:**

Gene expression of Survivin, CK19, CEA and VEGF was higher than in normal controls in 98.6%, 97.1%, 42.9% and 38.6% of cases, respectively, suggesting a potential diagnostic value of both Survivin and CK19. At multivariable survival analysis, TNM staging and Survivin mRNA levels were retained as independent prognostic factors, demonstrating that Survivin expression in the peripheral blood adds prognostic information to the TNM system. In contrast with previously published data, the transcript abundance of CEA, CK19 and VEGF was not associated with patients' clinical outcome.

**Conclusions:**

Gene expression levels of Survivin add significant prognostic value to the current TNM staging system. The validation of these findings in larger prospective and multicentric series might lead to the implementation of this biomarker in the routine clinical setting in order to optimize risk stratification and ultimately personalize the therapeutic management of these patients.

## Background

Gastric cancer represents the fourth most common cancer and second leading cause of cancer-related death worldwide. The estimated current incidence of gastric cancer is approximately 16.2/100 000 persons/year (world standardized rate, WSR), with highest incidences in Eastern Asia, Eastern Europe and South America [1]. At present the only prognostic system routinely employed for the management of gastric cancer patients is based on the International Union Against Cancer Tumor-Node-Metastasis (TNM) staging system [2], in which the degree of tumor penetration (pT) and nodal status (pN) [3] are the two main prognostic indicators in patients without distant metastatic disease. Patients in early stages are considered candidates for cure by surgery. However, 50% of gastric cancer patients suffer from tumor relapses even after radical surgery [4,5]. Thus, the current staging system does not seem to accurately predict individual patient risk of cancer recurrence. Indeed this classification identifies broad categories with significantly different prognostic subgroup within each stage, which make this system suboptimal for a personalized therapeutic approach. This is exemplified by the fact that some patients currently classified as "low-risk" are not submitted to adjuvant therapy, although they do experience disease relapse. Vice versa, some patients currently undergoing adjuvant therapy because of "high risk" TNM classification, would not need this treatment [6]. In order to address this issue and improve upon the prognosis of patients, new parameters reliably predicting patients' outcome are urgently needed. Patients who had undergone potentially curative surgeries retain the risk of recurrence that originates from microscopic tumor residues known as minimal residual disease (MRD). MRD can affect different body compartments, including the bone marrow, lymph nodes and peripheral blood [7]. In recent years, several studies have focused on the detection of circulating tumor cells (CTC) with respect to their clinical implications for patients with gastric cancer. As a result, quantitative real time polymerase chain reaction (qrtPCR) and variations of this technique, which is considered to be the most sensitive method for evaluating gene expression, have been used in the detection of tumor markers that indicate the presence of CTC in the blood [8].

In the current analysis, we aimed to profiling the peripheral blood of 70 patents affected with gastric adenocarcinoma by using qrtPCR. We tested four biomarkers, two of the presence, ad two of the aggressiveness of the tumor, and one of these, the Survivin, add independent prognostic power to the TMN staging system. This might allow for a better stratification of patient's risk and thus a better therapeutic management, especially in the adjuvant setting.

## Methods

### Patients

In this study we enrolled 70 patients (39 men, 31 women; age range 28 - 90 years, median age 68 years) who underwent surgery (total or partial gastrectomy) for histologically proven gastric carcinoma between October 1998 and November 2007, and for whom a peripheral blood sample was available. The study, which is in compliance with the Helsinki Declaration, was approved by the Local Ethical Committee of the University of Padova (approval number: 70/2006). Written informed consent regarding the use of biological specimens for investigational purposes was obtained from all patients. At the time of the analysis, 33 (47.1%) were alive whereas 37 (52.9%) had died. Median follow-up was 15 months (range: 6-119 months). Median survival was 25 months. Tumor characteristics are summarized in Table 1. The depth of tumor invasion (T category), extent of lymph node metastasis (N category) and macroscopic metastasis (M category) were categorized according to the UICC TNM staging system.

**Table 1:** Patients and tumor characteristics.

Parameters		N	(%)
Patients			
	all	70	(100.0)
	male	39	(55.7)
	female	31	(44.3)

Age (years)			
	≤ 65	21	(30.0)
	> 65	49	(70.0)

Location			
	multicentric	5	(7.1)
	upper third	11	(15.7)
	middle third	19	(27.1)
	lower third	35	(50.0)

TNM stage			
	I (IA + IB)	10	(14.3)
	II	14	(20.0)
	III (IIIA + IIIB)	19	(27.1)
	IV	27	(38.6)

### Cell lines

The human gastric carcinoma cell line NCI-N87, obtained from the American Type Culture Collection (Manassas, USA), was incubated in RPMI-1640 medium (Invitrogen - Gibco, Carlsbad, CA, USA) containing 10% fetal calf serum (Euroclone - Celbio, Pero, MI), 10 mM Hepes, 1 mM Sodium Pyruvate (Sigma-Aldrich, St. Louis, MO), at 37°C in 5% CO_2_.

NCI-N87 is a gastric carcinoma cell line derived from a liver metastasis of a well differentiated carcinoma of the stomach taken prior to cytotoxic therapy. According to the manufacturer's datasheet, cells express the surface glycoproteins carcinoembryonic antigen (CEA).

### Cell spiking experiments

A cell spiking study was performed in order to determine the sensitivity of this qrtPCR technique for detecting cancer cells in peripheral blood mononuclear cells (PBMC). The PBMC were obtained from healthy volunteers, and were counted and diluted 1:1 in RPMI medium. Gastric cancer cells N87 were counted and serially diluted from 1 × 10^6 ^cells/milliliter to 1 cell/milliliter in the PBMC. Total RNA was extracted and reverse- transcribed from 2 milliliters of each fraction. qrtPCR for two genes of interest were then performed, as described below.

### Sample collection, RNA extraction and cDNA synthesis

A 6 ml aliquot of venous blood was obtained from each patient at the time of surgery. Sample processing was performed within two hours after blood withdrawal. Sample was centrifuged at 2000 × g for 10 minutes, and the PBMC fraction was collected and stored in liquid nitrogen.

Frozen samples were thawed and total RNA was extracted using the Guanidinium Thiocyanate-Phenol-Chloroform method (Trizol Reagent, Invitrogen, Carlsbad, CA, US). The integrity of the isolated RNA was established by qrtPCR analysis of the endogenous reference gene beta actin (b-actin) as described below [9].

Total RNA (7 μg per 100 μl final reaction volume) was reverse-transcribed using random primers and MultiScribe Reverse Transcriptase (High-Capacity cDNA reverse transcription kit, Applied Biosystems, Foster City, CA, USA). The reaction mixture was incubated for 10 minutes at 25°C, then at 37°C for 120 minutes. cDNA was stored at -80°C until use.

### Real-time quantitative polymerase chain reaction

The transcriptional levels of four genes (i.e., carcinoembryonic antigen [CEA], cytokeratin-19 [CK19], Survivin and vascular endothelial growth factor [VEGF]) were measured in the peripheral blood of patients by means of quantitative real time PCR (qrtPCR) using the relative quantification method (2^-ΔΔCt ^method) [10,11]. Using the 2^-ΔΔCt ^method the data are presented as the fold-change in gene expression normalized by a reference gene and relative to a calibrator sample. The purpose of the normalization process is to adjust the value representing the transcriptional levels of each gene by the amount of mRNA present in each sample: this is usually obtained by dividing the expression levels of each gene of interest by those of a reference gene (also called "housekeeping" gene). Housekeeping genes are expressed constitutively and - ideally - are not affected by experimental manipulations: therefore, they should approximately reflect the total amount of mRNA tested in each sample [12]. As the reference gene in this study we used beta-actin, one of the most commonly used housekeeping genes.

In a relative quantification PCR study, the so called "calibrator" is represented by the gene expression of a chosen sample by which the expression profile of each sample of interest is adjusted: this approach enables the investigator to compare the expression profile of the samples of interest with each other in terms of their fold-change with respect to a single sample (the calibrator).

Usually, the calibrator is an untreated sample (e.g. in a functional study), a sample at time zero (e.g. in a time-course study) or any unrelated sample (e.g. healthy controls in a patients study, or normal fibroblasts in a cancer cell line study) [10]. A pool of cDNA derived from PBMC of 20 healthy donors was used as the calibrator source in our study. Evaluation of the 2^-ΔΔCt ^indicates the fold change in gene expression relative to the calibrator. For the calibrator the ΔΔCt equals zero, and 2^0 ^equals one, so that the fold change in gene expression relative to the calibrator equals one, by definition.

The method was validated for our experimental system by verifying that the efficiencies of amplification of the targets and the b-actin genes were similar. TaqMan Gene Expression Assays specific for CEA, CK19, Survivin and VEGF were purchased from Applied Biosystems. To avoid amplifying contaminated genomic DNA, the primer pair was placed at the junction between two exons. The qrtPCR assay was performed using the ABI PRISM 7300 Sequence Detection system. The PCR reaction proceed in a mixture (30 μl) containing 15 μl of 2× TaqMan Universal PCR Master Mix, 1.5 μl of 20× TaqMan Gene Expression assay (all reagents from Applied Biosystems), 12.5 μl of water and 1 μl of cDNA template. Fifty cycles of amplification were performed at 95°C (15 seconds) and 60°C (1 minute) and mRNA expression levels were normalized against quantified b-actin mRNA expression for each sample.

### Statistical analysis

Statistical analyses were performed using the Stat View V. 4.57 software (Abacus Concepts, London, UK) and the StatXact V7.0.0 software (Cytel Software Corporation, USA). The correlation between gene levels (high versus low gene expression, as defined by the median value) and disease TNM staging categories was assessed by using the Cochrane-Armitage trend test. Survival curves were estimated using the Kaplan-Meyer method, and univariate survival comparisons were calculated according to the log-rank test. The transcriptional levels of the four genes, along with anthropometric factors and TNM stages (according to the 5th edition of the AJCC TNM staging system released in 1997), were utilized as independent variables in the multivariate survival analysis, which was performed using the Cox proportional hazards regression model [13]. The selection of variables that significant contribute to the predictive model was performed by stepwise method. Probability values <5% were considered statistically significant.

## Results

### Cell spiking: sensitivity of qrtPCR technique

**Figure 1:**
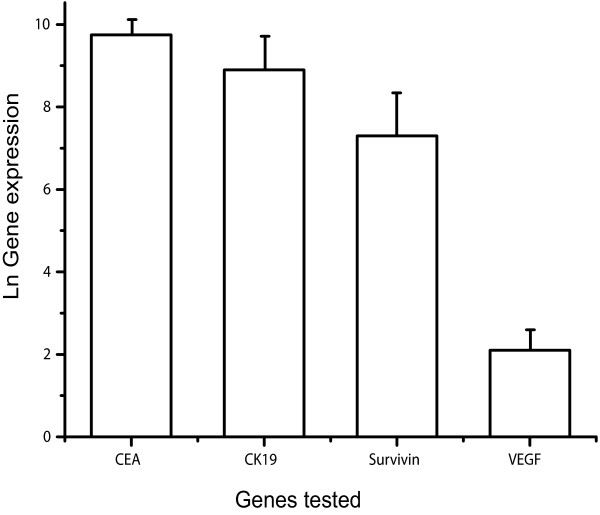
**Gene expression levels of the four genes of interest in N87 human gastric cancer cells (as measured by quantitative real time PCR: see text for details)**. The natural logarithm of the expression levels is reported on the y axis: the axis origin (0) represents the reference sample (called calibrator) with which all experimental samples are compared in the relative quantification method (see text for details).

In the N87 cell line, mRNA levels of the CEA, CK19, Survivin, and VEGF genes were highly expressed (Figure 1): therefore, this gastric carcinoma cell line represented a valid positive control for our experiments.

To establish the detection limit (sensitivity) of the qrtPCR technique, serial 10-fold dilutions (in PBMC) of N87 gastric carcinoma cells were assayed in triplicate by qrtPCR using CEA and Survivin as gene markers. CEA and Survivin mRNA were detected up to 1 cell/10^8 ^PBMC dilution: this corresponds to finding 1 to 10 malignant cells per 10 ml of peripheral blood (data not shown).

### Expression markers in blood samples

Peripheral blood samples from all 70 patients were evaluated for the four gene markers. The expression was positive (higher than calibrator) in 98.6%, 97.1%, 42.9%, 38.6% of samples for Survivin, CK19, CEA, VEGF, respectively (Table 2). Since Survivin and CK19 gene levels found in nearly all patients are greater than those in healthy controls, these findings point to a diagnostic potential of both genes.

**Table 2:** Transcriptional levels of four prognostic markers in the peripheral blood of patients with gastric cancer.

Marker	Above calibrator (%)*	Below calibrator (%)*	Undetectable (%)
Survivin	69 (98.6)	1 (1.4)	0 (0.0)

CK19	68 (97.1)	2 (2.9)	0 (0.0)

CEA	30 (42.9)	21 (30.0)	19 (27.1)

VEGF	27 (38.6)	43 (61.4)	0 (0.0)

* Gene expression levels were measured by quantitative real time PCR: using the relative quantification method, samples were classified as above or below the levels found in the calibrator (pooled peripheral blood samples from healthy donors).

**Figure 2:**
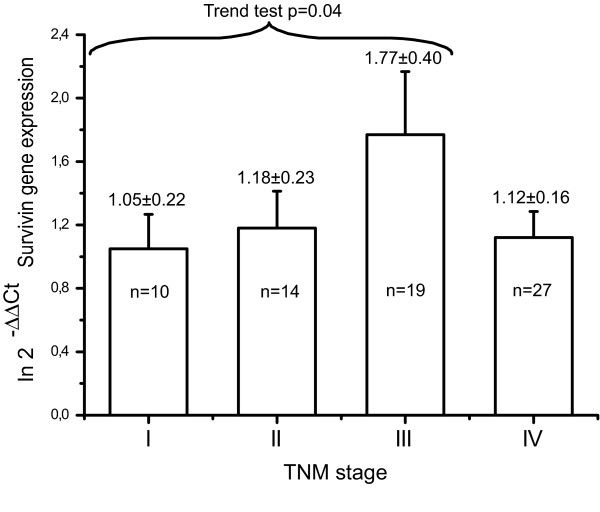
**Survivin gene levels measured in the peripheral blood of 70 patients with TNM stage I to IV gastric cancer**. The trend test analysis shows a significant increase in Survivin transcriptional levels across patients with stage I to III gastric cancer (trend test P-value = 0.04). For each column is reported the mean ± SD.

Moreover, if we considered the 75th percentile of Survivin expression levels per stage, we had a significant increased trend (p = 0.04) for stages from I to III, but not for stage IV (Figure 2).

### Survival analysis

**Figure 3:**
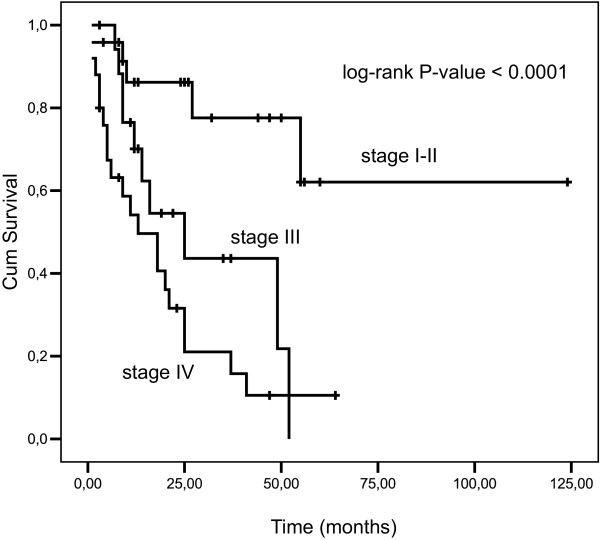
**Overall survival for TNM stage I-II, III and IV for all patients (log-rank test P-value < 0.0001)**.

After a mean follow up of 26 months, the median overall survival (OS) for TNM stage I-II was not reached; the median OS for stage III and IV was 25 and 13 months, respectively (log-rank test P-value < 0.0001, Figure 3).

**Figure 4:**
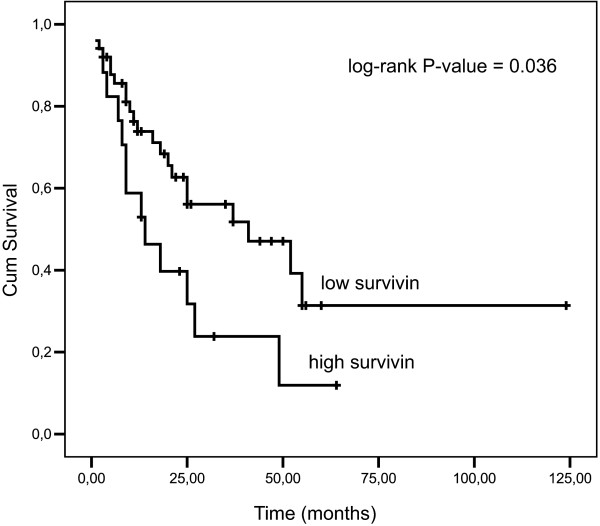
**Kaplan-Meier survival curves of patients with high (> 75th percentile) or low (< 75th percentile) transcriptional levels of Survivin measured in the peripheral blood**. Log-rank test P-value = 0.036.

Upon univariate survival analysis, among the four genes we have tested, only Survivin expression could identify two patient groups with significantly different prognosis. In fact, considering the 75th percentile of log-transformed gene transcription levels (1.508) as the cutoff point, median OS for high (>1.508) and low (≤1.508) Survivin mRNA abundance were 14 and 41 months, respectively (log-rank P-value = 0.036, Figure 4).

The multivariate survival analysis including TNM stage, age, gender and mRNA levels of the four markers showed that only TNM stage and Survivin mRNA levels measured in the peripheral blood independently predict patients' OS. The hazard ratio (HR) associated with Survivin levels indicates the increase in risk of death for 100 fold increase in Survivin expression (Table 3).

**Table 3:** Multivariate survival analysis of 70 patients with gastric cancer.

Covariates	HR	95% CI		P-value
		*Lower limit*	*Upper limit*	
TNM stage I	1 (reference)	-	-	-

stage II	1.20	1.01	1.45	0.048

stage III	1.34	1.05	1.70	0.017

stage IV	3.17	1.38	6.77	0.004

Survivin*	1.34	1.14	1.53	< 0.001

HR: hazard ratio; CI: confidence interval; * the HR associated with Survivin levels indicates the increase of death risk for 100-fold increase in Survivin expression.

Since only these four independent variables were retained by the Cox model, this analysis suggests that Survivin gene expression can add useful prognostic information to well established factors such as the TNM staging system.

## Discussion

In this study we found that transcriptional levels of Survivin measured in the peripheral blood of patients with gastric carcinoma independently correlate with their overall survival.

If validated in larger prospective studies, these results would allow to increase the prognostic power of conventional prognostic factors, which are currently embodied by the TNM staging parameters. This is of special relevance for patients with TNM stage I to III disease, for whom optimal risk stratification is essential to identify subjects with the highest likelihood to benefit from adjuvant treatments.

Our findings are of particular relevance also from the tumor biology viewpoint because the Survivin gene encodes a key anti-apoptotic protein belonging to the inhibitor of apoptosis protein (IAP) family. Beside being one of the best characterized anti-apoptotic factors [14], Survivin is the object of intense investigation due to the fact that in adults it is selectively expressed virtually only by cancers of different origin; moreover, its expression in the primary tumor has been associated with worse prognosis and resistance to conventional chemotherapeutics [15]. These observations make Survivin an ideal target for tumor-specific therapies, such as small molecule inhibitors and antigen-specific immunotherapy [16].

Survivin protein expression in primary tumors, including gastric cancer, has been investigated as a prognostic factor, higher levels being associated with worse cancer outcome [17]. About RNA expression, Survivin mRNA levels have been investigated as a marker of circulating tumor cells (CTC) in different kind of cancer, but the available data are scarce [18,19]. In a series of 26 gastric cancer patients, Survivin mRNA (as measured by means of ELISA-based qrtPCR) in the peripheral blood has been reported to correlate with patients' prognosis, the TNM staging being excluded from the final mutivariable model (forcing all variables into the Cox model) [20]. In our larger series (n = 70), the prognostic role of Survivin blood levels is confirmed, although the TNM staging remains a significant prognostic factor in the final multivariable model (using the stepwise mode for variable selection). Despite the significant association with patients' prognosis, Survivin mRNA levels did not increase across all four TNM stages: the trend for increased transcriptional abundance was in fact demonstrated only in patients with stage I to III disease (Figure 2). This finding might depends upon the low sample sizes of the single TNM stages (and the consequent low statistical power) but might also indicates that Survivin plays a role in locoregional and not in distant metastatic disease (where other genes could be more relevant, as recently reported [21]).

Unlike other studies [9,22-26], the levels of CK19 and CEA did not correlate with patients survival in our series, either on univariable (data not shown) or multivariable survival analysis: this might depend upon several factors. First of all, the inclusion in our multivariable analysis (with a stepwise mode of variable selection) of a marker not considered by other Authors makes any comparison unfeasible.

Of note, some positive reports only use univariable survival analysis, which jeopardizes the reliability and reproducibility of their results, once adjustment for well established prognostic factors (i.e. TNM stages) were implemented [9,25,27,28]. Moreover, in line with our results other investigators do report lack of association between cytokeratins positive cells presence and prognosis [29] (Table 4).

**Table 4:** Selected series analyzing the prognostic role of circulating tumor cells (CTC) in patients with gastric cancer.

Author	Year	Ref.	Patients	Method	Survival analysis	Markers	Findings
Koga T et. al.	2008	[9]	101	Quantitative RT-PCR	Univariate	CK18, CK19, CK20, CEA	CK19 is the better marker, and is usable to estimate prognosis or for adjuvant treatment

Yie SM et. al.	2008	[20]	26 (gastric cancer)	RT-PCR ELISA	Multivariate	Survivin	Status of Survivin-expressing CTC is a strong and independent predictor for recurrence

Hiraiwa K et. al.	2008	[22]	44 (gastric cancer)	CellSearch system	Multivariate	CD45 (-) cells vs CK (+) cells	CTSs significantly correlated with advanced tumor stage

Illert B et. al.	2005	[23]	70	Quantitative RT-PCR	Multivariate	CK20	CK20 is an independent prognostic marker

Yeh KH et. al.	1998	[24]	34	Nested quantitative RT-PCR	Univariate	CK19	CK19 expressing CTC are associated with poor prognosis

Seo JH et. al.	2005	[25]	46	Quantitative RT-PCR	Not performed	CEA	CEA mRNA is significantly correlated with clinical recurrence

Wu CH et. al.	2006	[26]	42	Quantitative RT-PCR	Not performed	hTERT, CK19, CK20, CEA	CEA mRNA is correlated with higher risk of postoperative recurrence/metastasis

Uen YH et. al.	2006	[27]	52	Quantitative RT-PCR	Univariate	c-MET, MUC1	c-Met and MUC1mRNA significantly correlate with prognosis

Mimori K et. al.	2008	[28]	810	Quantitative RT-PCR	Univariate	MT1-MMP	MT1-MMP is an independent factor for determining recurrence and distant metastasis

Pituch-Noworolska A et. al.	2007	[29]	57	Flow cytometry	Univariate	CD45 (-) cells vs CK (+) cells	The presence of CK (+) cells is of no prognostic value

These conflicting data might depend on the fact that CK19 and CEA are markers of CTC presence but not necessarily of CTC ability to metastasize: in fact, it is well accepted that only a subset of CTC has the biological potential of giving rise to metastatic deposits, while most CTC ultimately die without being harmful for the host [7]. Accordingly, markers of CTC "aggressiveness" such as Survivin might reveal to be more informative in terms of correlation with patients' prognosis.

Of note, in the present study the mRNA abundance of Survivin and CK19 was always greater than that found in healthy controls except for one and two cases, respectively. This underscores the importance of using a quantitative method (qrtPCR) that enabled us to stratify patients risk on a continuous scale, whereas standard PCR would have classified virtually all patients as positive. On the other side, this finding - which to the best of our knowledge has never been reported before - suggests that these genes might be exploited also for diagnostic purposes, although a dedicated study specifically designed for this aim is warranted.

As a final consideration, we would like to observe that PCR-based methods do not allow to identify the cell source of the measured markers: in fact, all these methods require the lysis of the cells harvested from the peripheral blood of patients in order to extract the mRNA used to assess the expression of target genes. Besides CTC, other potential sources of PCR-detected genes are PBMC, circulating endothelial cells (CEC), bone marrow derived circulating stem cells as well as skin cells (e.g. keratinocytes, fibroblasts, melanocytes) contaminating the sample during blood withdrawal. Nevertheless, CTC are likely to be the principal cell source for Survivin as its expression is very limited in normal adult tissues and is instead mainly restricted to malignant cells [30]. In this regard, cytometric methods are less prone to false positive results, as they imply antibody-based cell sorting followed by cytological identification of tumor cells that precedes their phenotypic characterization [31].

## Conclusions

Gene expression levels of Survivin add significant prognostic value to the current TNM staging system of patients with gastric carcinoma. The validation of these findings in larger prospective series might lead to optimize the risk stratification and ultimately to personalize the therapeutic management of these patients.

## Competing interests

The authors declare that they have no competing interests.

## Authors' contributions

LB conceived the study design, handled biological samples, performed qrtPCR analysis and drafted the manuscript. SM conceived the study design, performed statistical data analysis and drafted the manuscript. JG, AM and PP participated in the design of the study and collected the clinical data of patients. RS handled samples collection and storage until RNA extraction. DN coordinated the study and participated in manuscript writing and editing. All authors read and approved the final version of the manuscript.
